# Investigating oral somatosensory perception and oral symptoms of head and neck cancer patients: insights on eating behaviour

**DOI:** 10.1007/s00520-024-08512-4

**Published:** 2024-05-01

**Authors:** Reisya Rizki Riantiningtyas, Anestis Dougkas, Wender L. P. Bredie, Camille Kwiecien, Amandine Bruyas, Pierre Philouze, Agnès Giboreau, Florence Carrouel

**Affiliations:** 1 Institute Lyfe (Formerly Institut Paul Bocuse) Research Centre, Chateau Du Vivier, BP 25 - 69131 Ecully Cedex, France; 2 Health Systemic Process (P2S), Research Unit UR4129, University of Lyon, University Claude Bernard Lyon 1, 69008 Lyon, France; 3 Section for Food Design and Consumer Behaviour, Department of Food Science, Faculty of Science, University of Copenhagen, 1958 Frederiksberg C, Denmark; 4 Danone Global Research & Innovation Center, 3584 CT Utrecht, The Netherlands; 5 Institute of Cancerology, Hôpital Croix Rousse, Hospices Civils de Lyon, 69004 Lyon, France; 6 Laboratoire Centre Européen Nutrition Et Santé (CENS), Université Claude Bernard Lyon 1, 106069310 CarMeNPierre-Bénite, Unité INSERM France; 7 ORL Service and Cervico-Facial Surgery, Hospices Civils de Lyon, 69004 Lyon, France

**Keywords:** Oral somatosensory perception, Sensory alteration, Oral symptoms, Head and neck cancer, Questionnaire, Eating behaviour

## Abstract

**Purpose:**

Sensory alterations and oral manifestations are prevalent among head and neck cancer (HNC) patients. While taste and smell alterations have been thoroughly investigated, studies on their oral somatosensory perception remain limited. Building upon our previous publication that primarily focused on objective somatosensory measurements, the present work examined self-reported sensory perception, including somatosensation and oral symptoms, in HNC patients and evaluated their link with eating behaviour.

**Methods:**

A cross-sectional study was conducted using self-reported questionnaires on sensory perception, oral symptoms, sensory-related food preference, and eating behaviour among HNC patients (*n* = 30). Hierarchical clustering analysis was performed to categorise patients based on their sensory perception. Correlations between oral symptoms score, sensory perception, sensory-related food preference, and eating behaviour were explored.

**Results:**

Two distinct sensory profiles of patients were identified: no alteration (*n* = 14) and alteration (*n* = 16) group. The alteration group showed decreased preference towards several sensory modalities, especially the somatosensory. Concerning eating behaviour, more patients in the alteration group agreed to negatively connotated statements (e.g. having food aversion and eating smaller portions), demonstrating greater eating difficulties. In addition, several oral symptoms related to salivary dysfunction were reported. These oral symptoms were correlated with sensory perception, sensory-related food preference, and eating behaviour.

**Conclusion:**

This study presented evidence demonstrating that sensory alterations in HNC patients are not limited to taste and smell but cover somatosensory perception and are linked to various aspects of eating. Moreover, patients reported experiencing several oral symptoms. Those with sensory alterations and oral symptoms experienced more eating difficulties.

**Supplementary Information:**

The online version contains supplementary material available at 10.1007/s00520-024-08512-4.

## Introduction

Altered eating, or “losing the ability to eat well”, is a problem among cancer patients, including and especially among head and neck cancer (HNC) patients due to the cancer location being in the food ingestion site [1–3]. Several side effects were reported prior to, during, and following cancer treatments that interfere with their eating ability. These oral symptoms include dry mouth, chewing/swallowing difficulty, and mucositis [4, 5]. The symptom of dry mouth is reported by up to 90% of HNC patients following radiotherapy, which affects their ability to eat since a reduction in saliva alters the formation of the food bolus and makes swallowing difficult [5, 6]. Mucositis, experienced by 80–90% of HNC patients, induces pain and oral discomfort, which also affects their ability to eat [5, 7]. These oral symptoms lead to an alteration in eating habits that reduce food intake and may contribute to a decline in nutritional status [5, 8]. For instance, 51–74% of HNC patients were malnourished [2, 3]. It also adds a psychological burden as patients lose pleasure from eating and the social interactions surrounding mealtimes [9]. Consequently, this leads to a lowered quality of life [10].

Another aspect that contributed to the altered eating experience was the sensory aspect. Altered sensory perception plays a crucial role in cancer patients’ eating behaviour. Eating behaviour is a broad and complex term encompassing aspects of eating that can influence individuals’ nutritional choices. This includes food choices (preferences and avoidance), food intake, and eating experience [11]. It was shown that sensory alteration was correlated to lower energy intake and higher weight loss, contributing to declined nutritional status and quality of life of advanced HNC patients and patients with gastrointestinal stromal tumours [10, 12].

Several studies have investigated sensory alterations among HNC patients, focusing on alterations in smell and taste perception, with the prevalence ranging from 30 to 80% [13, 14]. However, sensory perception is not only limited to taste and smell but also somatosensation, which few studies have investigated [1, 15, 16]. Somatosensation comprises perception towards texture, temperature, and chemesthetic sensations (e.g. spiciness of chilli and cooling sensation of peppermint) processed by the trigeminal system [17]. In addition, overall food perception is highly dependent on the oral condition, such as salivary function and oral health status. For instance, it was shown that saliva influences the perception of food texture [18].

HNC patients reported oral complaints such as sensitivity to texture, sensitivity to spices, dry mouth, mucositis, and difficulty in chewing or swallowing [1, 15]. These oral complaints are related to somatosensory aspects and oral symptoms. HNC patients also reported that these changes have led to adjustments in their diets, such as adding sauce/gravy to add extra moisture to the food, blending the food, and avoiding certain foods with difficult textures (e.g. dry bread, red meat, hard vegetables) [16]. To better understand their eating experience, it is necessary to assess not only their taste and smell perception but also their somatosensory perception and oral symptoms. In contrast to the objective evaluation of somatosensation and salivary measures detailed in the previous part of our study [19], the present investigation delved into the self-reported sensory perception and oral symptoms of HNC patients, and their association with eating behaviour. It was hypothesised that sensory alterations among HNC consist not only of taste and smell alterations but also somatosensory alterations along with oral symptoms, altogether related to modified food preferences and eating behaviour.

## Materials and methods

### Study design

This questionnaire-based study was a part of the cross-sectional study (Somestalim study) registered to the Clinical Trials Registry (NCT05272917), conducted in accordance with the Declaration of Helsinki. The protocol and study design were approved by the Ethics Personal Protection Committee of Ile-de-France (RCB N° 2021-A02961-40). Informed consent was obtained from all participants. The Somestalim study is a cross-sectional study comparing HNC patients and matched control. The study consisted of objective measurements of salivary function and somatosensory sensitivity, as well as subjective measurements through self-reported questionnaires. The first part of the study, which focused on the objective measurements of the somatosensory perception of HNC patients compared to matched control, was reported in a previous publication by our group [19]. The present paper explores the subjective perception of HNC patients and its relationship with food preference and eating behaviour.

### Participants

Clinical research associates or physicians recruited thirty HNC patients during their outpatient consultations at the Hospices Civils de Lyon (France). Patients were individuals aged 18–70 years who had been diagnosed with tumours in the upper aerodigestive tract (including the oral cavity, pharynx, and larynx), salivary glands, maxillary sinuses, or nasopharynx. Additionally, patients have completed radiotherapy between 4 months and 5 years ago, as a standalone treatment or in combination with surgery and/or systemic treatment. Exclusion criteria were pregnant or breastfeeding individuals, having food allergy or intolerance, unable to swallow soft food, having restricted mouth opening (trismus), having difficulties extending the tongue, and having large tongue resection [19]

### Procedure

The study was conducted at Croix Rousse and Lyon-Sud hospitals from May 2022 to April 2023 (between 10.00 and 14.00). The exact time and location depended on the participant’s availability. Participants completed the questionnaires using a tablet, and data was collected via an online platform, Qualtrics (Provo, USA). The researcher was present to address any clarifying inquiries. The questionnaires took approximately 20 min to complete.

The self-reported questionnaires (Supplementary material S1) were developed specifically for the study and adapted from existing questionnaires [10, 20–24]. The questionnaire was developed in English and translated into French. Native speakers checked and verified the translations with the English questionnaire. The questionnaires were pilot-tested with healthy individuals (internal staff of the Institut Lyfe Research Centre) (*n* = 16) and cancer patients (*n* = 4) to ensure clarity. Following this step, the comments of the testers were considered, and the research team validated the final questionnaire.

The questionnaire included questions on sociodemographic (sex, age, country of residence). Furthermore, questions on sensory perception and sensory-related food preference [10, 20–22], oral symptoms [24], and eating behaviour [23] were included. The different parts of the questionnaires were as follows:

1. Sensory perception: The question started with a general question on taste: “I notice changes in the taste of food/drinks” with response options “1 = strongly disagree” to “6 = strongly agree”, followed by the individual evaluation on the different sensory modalities. Fourteen items covered five subsections including the basic tastes, smell, texture, temperature, and chemesthetic sensations. The questions were phrased as follows: “Compared to the situation before cancer treatment, I perceive that my *sensitivity* towards [salty/ sweet/ sour/ bitter/ umami/ smell of/ texture of/ cold/ hot/ pungent/ cooling/ astringent/ carbonated/ alcoholic] food/drink …”. The response options were: “has decreased/ remains unchanged/ has increased”, except for smell in which the response options were “has decreased/ remains unchanged/ has increased/ is different” and texture limited to “changed/ remains unchanged”.
2. Sensory-related food preference: Similar to the questions on sensory perception, the nine questions for sensory preference were phrased “In comparison with the situation before cancer treatment, my *preference* towards [sensory modality] food/ drink has..”. The response options were: “has decreased/ remains unchanged/ has increased”.
3. Eating behaviour: 15 statements related to eating behaviour with response options of “1 = disagree completely” to “6 = agree completely”.
4. Oral symptoms: 19 different oral symptoms with response options ranging from “1 = Never” to “5 = Always”.

### Data analysis

Descriptive statistics were used to describe the sociodemographic and clinical information of the participants. In order to explore the various sensory profiles of the patients, a clustering analysis was conducted based on their responses to sensory perception. The analysis involved two-way hierarchical clustering using Ward’s method, and the resulting heatmap was created using the *pheatmap* package (R package version 1.0.12) in R studio (version 4.3.1) and the code was registered in GitHub repository [25]. Chi-square test was used to compare the categorical data between the groups.

To investigate the relationship between oral symptoms and other variables, the scores for each of the 19 individual oral symptoms were added to create an oral symptom score. Sensory-related food preference was treated as a categorical variable with three levels: decreased, no change, and increased preference. Correlations between oral symptoms score, sensory perception, sensory-related food preference, and eating behaviour were assessed using the Spearman correlations. A *p*-value of ≤ 0.05 was considered significant. SPSS Statistics 23 (IBM Corporation) was used for statistical analyses.

## Results

### Characteristics of the study population

The complete demographic and clinical characteristics of patients are presented in Table 1. In total, 30 patients (23 males and 7 females, mean age 59.9 ± 7.5) diagnosed with tumour on the oropharynx, hypopharynx, nasopharynx, larynx, or oral cavity participated in the study. All patients received radiotherapy; 70% had surgery, and 47% had chemotherapy.

**Table 1:** Demographic and clinical characteristics of patients^a^ [19]

Variable	Patient (*n* = 30)
Age (mean ± SD)	59.9 ± 7.5
Sex	
*Male*	23 (77)
*Female*	7 (23)
Household	
*Alone*	6 (20)
*Living with partner/children*	23 (7)
*Other*	1 (3)
Smoking status	
*Current smoker*	6 (20)
*Former smoker*	4 (13)
*Non-smoker*	20 (67)
Alcohol consumption	
≥ *4* × */week*	3 (10)
*2–3* × */week*	9 (30)
*2–4* × */month*	7 (23)
≤ *1* × */month*	3 (10)
*Never*	8 (27)
Clinical characteristics	
Primary tumour site	
*Oropharynx*	17 (57)
*Hypopharynx*	2 (7)
*Nasopharynx*	2 (7)
*Oral cavity*	6 (20)
*Larynx*	3 (10)
Histologic type	
*Squamous cell carcinoma*	26 (87)
*Other*	4 (13)
Tumour stage	
*I*	0 (0)
*II*	3 (10)
*III*	13 (43)
*Iva*	9 (30)
*IVb*	2 (7)
*N/a*	3 (10)
Types of treatment	
*Radiation*	2 (7)
*Radiation* + *surgery*	14 (47)
*Radiation* + *surgery* + *systemic treatment*	7 (23)
*Radiation* + *systemic treatment*	7 (23)
Duration since the end of radiotherapy	
< *1 year*	11 (37)
> *1 year*	19 (63)

^a^The sum of percentages may be dissimilar to 100% due to rounding

### Sensory alterations among head and neck cancer patients

#### Patient clustering based on perceived sensory alterations

**Fig. 1:**
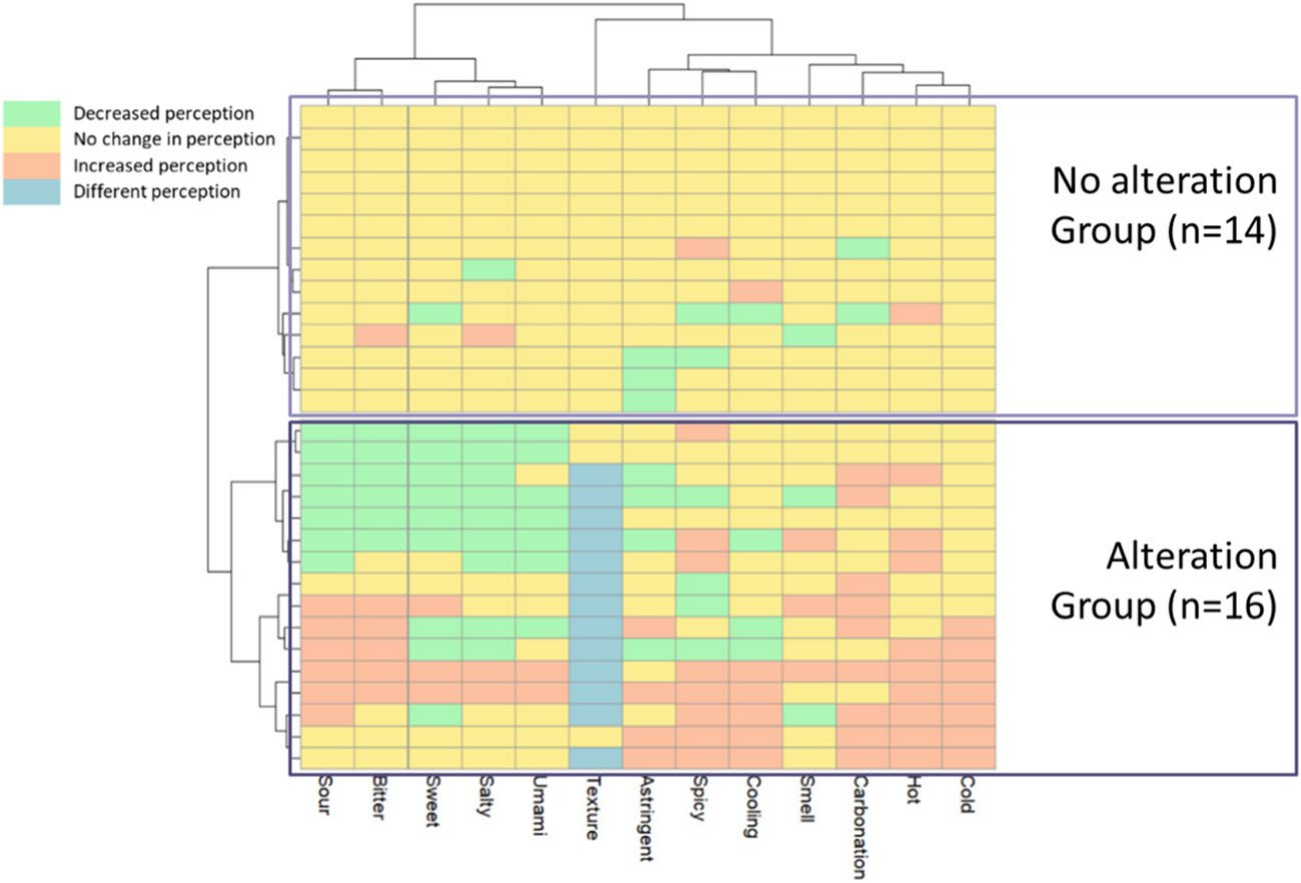
Heat-map diagram of a two-way hierarchical clustering analysis consisting of sensory perception of cancer patients. Questions were “In comparison with the situation before cancer treatment, I perceive that my sensitivity towards [sensory modality] food/drink has …” with response options of “increased/ not changed/ decreased/ changed”. Each row represents a patient, and each column represents their perception of each sensory modality

Hierarchical clustering allows patients to be classified based on their response to 14 items of the sensory perception questions (the “Procedure” section, questionnaire 1). Figure 1 illustrates that two distinct clusters were identified: (1) group of patients who did not perceive any alterations or perceived few alteration across the different sensory modalities (*n* = 14), hereafter mentioned as the “no alteration group” and (2) a group of patients with perceived alteration in several sensory modalities (*n* = 16), hereafter mentioned as the “alteration group”. Within the alteration group, some patients experienced increased sensitivity, decreased sensitivity, and a mixture of increased and decreased sensitivity across the different sensory modalities. The distribution of patients with/without chemotherapy (*p* = *0*.509) and duration since radiotherapy (*p* = 0.150) did not differ between the two groups.

#### Relationship between perceived sensory alteration and food preference

Based on the clustering, the two groups were first compared regarding their sensory-related food preference. Patients in the alteration group demonstrated significant differences in their sensory-related food preference compared to the no-alteration group (Table 2).

**Table 2:** Distribution of responses between groups in terms of sensory-related food preference

	No alteration (*n* = 14)	Alteration (*n* = 16)	*p*-value
Salty food products			
*Decreased preference*	1	2	0.310
*No change*	10	7	
*Increased preference*	3	7	
Sweet food products			
*Decreased preference*	2	6	0.152
*No change*	11	7	
*Increased preference*	1	3	
Sour food products			
*Decreased preference*	1_a_	8_b_	**0.011**
*No change*	13_a_	8_b_	
*Increased preference*	0_a_	0_a_	
Bitter food products			
*Decreased preference*	1_a_	8_b_	**0.028**
*No change*	12_a_	8_b_	
*Increased preference*	1_a_	0_a_	
Spicy food products			
*Decreased preference*	0_a_	9_b_	**< 0.001**
*No change*	14_a_	5_b_	
*Increased preference*	0_a_	2_a_	
Cooling food products			
*Decreased preference*	0_a_	7_b_	**0.013**
*No change*	13_a_	9_b_	
*Increased preference*	1_a_	0_a_	
Astringent food products			
*Decreased preference*	3_a_	12_b_	**0.003**
*No change*	11_a_	4_b_	
*Increased preference*	0_a_	0	
Carbonated beverages			
*Decreased preference*	1_a_	6_b_	**0.007**
*No change*	13_a_	6_b_	
*Increased preference*	0_a_	4_b_	
Alcohol			
*Decreased preference*	3_a_	10_b_	**0.024**
*No change*	11_a_	6_b_	
*Increased preference*	0_a_	0_a_	

The different subscript letters denote a significant difference between columns at the 0.05 level on the chi-square test. The significant *p*-values are emphasised in bold

While most patients in the no alteration group reported an unchanged preference compared to before their treatment, the alteration group showed a higher frequency of patients with a decreased preference towards sour (*p* = 0.011) and bitter (*p* = 0.028) tastes. For the alteration group, half of the patients reported a decreased preference for sour and bitter food, yet only one patient from the no alteration group reported a decreased preference. In addition, the two groups also significantly differed in their preference towards all somatosensory sub-modalities. More patients in the alteration group reported a decreased preference for spicy, cooling and astringent food products, as well as carbonated and alcoholic beverages.

#### Relationship between perceived sensory alteration and eating behaviour

Some differences between the two groups were also observed in their responses towards eating behaviour questions (Table 3). Higher proportions of patients agreed to negatively-connotated items such as eating smaller portions (*p* = 0.012), eating becomes effortful (*p* = 0.002), food aversion (*p* = 0.006), and certain food has become unpleasant/difficult to eat (*p* = 0.035).

**Table 3:** Distribution of responses between groups in terms of eating behaviour

Statements	No alteration group (*n* = 14)	Alteration group (*n* = 16)	*p*-value
Feeling hunger when smelling/seeing food			
*Disagree*	0_a_	5_b_	**0.031**
*Agree*	14_a_	11_b_	
Eating a variety of food			
*Disagree*	3	8	0.107
*Agree*	11	8	
Trying novel food			
*Disagree*	2	6	0.154
*Agree*	12	10	
Having less appetite			
*Disagree*	8	8	0.491
*Agree*	6	6	
Feeling satiated quickly			
*Disagree*	7	9	0.509
*Agree*	7	7	
Eating smaller portion			
*Disagree*	11_a_	5_b_	**0.012**
*Agree*	3_a_	11_b_	
Eating more frequently			
*Disagree*	9	11	0.550
*Agree*	5	5	
Eating becomes demanding/effortful			
*Disagree*	11_a_	4_b_	**0.002**
*Agree*	3_a_	12_b_	
Losing eating pleasure			
*Disagree*	10	7	0.123
*Agree*	4	9	
Not feeling at ease when eating out			
*Disagree*	12	10	0.154
*Agree*	2	6	
Being the last to finish meal			
*Disagree*	4	5	0.596
*Agree*	10	11	
Disliking food before tasting			
*Disagree*	10	10	0.450
*Agree*	4	6	
Having food aversion			
*Disagree*	13_a_	7_b_	**0.006**
*Agree*	1_a_	9_b_	
Having food craving			
*Disagree*	6	8	0.491
*Agree*	8	8	
Certain food has become unpleasant			
*Disagree*	9_a_	4_b_	**0.035**
*Agree*	5_a_	12_b_	

The different subscript letters denote a significant difference between columns at the 0.05 level on the chi-square test. The significant *p*-values are emphasised in bold

### Oral symptoms of head and neck cancer patients

Oral symptoms frequently experienced by more than 50% of the patients include dry mouth (80%), difficulty swallowing (67%), sticky saliva (60%), difficulty chewing (57%), food stuck in the throat (57%), and food stuck in the mouth (53%) (Table 4). Other oral symptoms that were frequently experienced were dental problems, sensitive teeth/gum, and pain in the throat.

**Table 4:** Reported oral symptoms, *n* (%)

Oral symptoms	Never	Rarely	Sometimes	Often	Always	Subtotal^a^
Dry mouth	1 (3)	5 (17)	6 (20)	11 (37)	7 (23)	24 (80)
Difficulty swallowing	7 (23)	3 (10)	10 (33)	8 (27)	2 (7)	20 (67)
Sticky saliva	8 (27)	4 (13)	6 (20)	8 (27)	4 (13)	18 (60)
Difficulty chewing	6 (20)	7 (23)	9 (30)	6 (20)	2 (7)	17 (57)
Food stuck in the throat	11 (37)	2 (7)	10 (33)	6 (20)	1 (3)	17 (57)
Food stuck in the mouth	11 (37)	3 (10)	10 (33)	4 (7)	2 (7)	16 (53)
Avoiding certain food due to dental problem	16 (53)	1 (3)	8 (27)	1 (3)	2 (7)	13 (43)
Sensitive teeth/gum	11 (37)	6 (20)	3 (10)	7 (23)	3 (10)	13 (43)
Pain in throat	14 (47)	3 (10)	6 (20)	6 (20)	1 (3)	13 (43)
Pain/problem with teeth	17 (57)	0 (0)	5 (17)	6 (20)	2 (7)	13 (43)
Fear of eating due to pain	18 (60)	2 (7)	5 (17)	3 (10)	2 (7)	10 (33)
Painful mouth	18 (60)	3 (10)	4 (7)	3 (10)	2 (7)	9 (30)
Oral inflammation	12 (40)	9 (30)	9 (30)	0 (0)	0 (0)	9 (30)
Pain in gum	17 (57)	5 (17)	5 (17)	2 (7)	1 (3)	8 (27)
Trismus	18 (60)	5 (17)	4 (7)	1 (3)	2 (7)	7 (23)
Burning sensation in the mouth	19 (63)	4 (7)	2 (7)	2 (7)	3 (10)	7 (23)
Bleeding gum	21 (70)	4 (7)	4 (7)	1 (3)	0 (0)	5 (17)
Painful lips	22 (73)	3 (10)	3 (10)	1 (3)	1 (3)	5 (17)
Nausea	18 (60)	7 (23)	4 (7)	1 (3)	0 (0)	5 (17)

^a^Subtotal to the frequency of sometimes, often, and always for each symptom

Correlations between oral symptom score and other variables, including sensory perception, sensory-related food preference, and eating behaviour, were explored (Supplementary Figs. 1, 2 and 3). Oral symptom scores showed moderate positive correlations with changes in texture (*r* = 0.54, *p* = 0.002) and temperature (*r* = 0.56, *p* = 0.001 for hot and *r* = 0.42, *p* = 0.021 for cold) perception. In particular, these changes in perception were correlated to oral symptoms such as difficulty in chewing and swallowing, sensitive teeth/gums, and pain surrounding the oral cavity.

The oral symptom score also showed negative correlations with a preference towards sour (*r* =  *− *0.51, *p* = 0.004), spicy (*r* =  *− *0.51, *p* = 0.004), carbonated (*r* =  *− *0.45, *p* = 0.012), and astringent (*r* =  *− *0.46, *p* = 0.012) food products, and alcohol (*r* =  *− *0.39, *p* = 0.036). In particular, this decline in preference was correlated with oral symptoms such as difficulty swallowing, food getting stuck in the throat/mouth, dry mouth, oral inflammation, and pain surrounding the oral cavity.

Regarding eating behaviour, the oral symptom score was negatively correlated with consuming a variety of foods (*r* =  *− *0.48, *p* = 0.007), in particular, driven by difficulty swallowing, food stuck in the throat, and pain surrounding the oral cavity. Meanwhile, oral symptom score was positively correlated with having less appetite (*r* = *0*.42, *p* = 0.002), eating smaller portions (*r* = 0.49, *p* = 0.006), effortful eating (*r* = 0.54, *p* = 0.002), losing pleasure in eating (*r* = 0.43, *p* = 0.019), feeling discomfort when eating out (*r* = 0.53, *p* = 0.002), not liking food before tasting (*r* = 0.44, *p* = 0.015), developing food aversion (*r* = 0.66, *p* < 0.001), and food becoming unpleasant or difficult to eat (*r* = 0.65, *p* < 0.001).

## Discussion

More than half of the HNC patients in this study reported experiencing sensory alterations, which is in agreement with the prevalence of self-reported sensory alteration ranging between 12 and 84% among various cancer patients [11]. Among these HNC patients, changes in taste and somatosensory perception (texture, temperature, and chemesthesis) were reported more frequently than changes in smell perception (Fig. 1), which is consistent with earlier observations [26]. A study showed that changes in smell perception tend to be gradual and unnoticed compared to taste perception [27].

The study highlights the relationship between sensory alteration, sensory-related food preference, and eating behaviour. Upon categorising the patients into two distinct profiles, the alteration group demonstrated a higher proportion of patients with a reduced preference for all somatosensory sub-modalities as well as towards bitter and sour tastes. These findings suggest that changes in sensory perception are linked with sensory-related food preferences. Similar observations have been reported in previous studies [20, 28]. Among patients receiving anti-tumour therapy, significant differences in product preferences of various oral nutritional supplements (ONS) were observed in the patient group with altered perception but not in the unaltered perception group [21]. Among testicular cancer patients, taste and smell sensitivity was associated with liking of ONS [28]. Further, previous studies showed that sensory alteration was related to reduced appetite, food appreciation, and food selection or intake [29–31].

The presence of sensory alteration was also reflected in items concerning eating behaviour. A higher proportion of patients with sensory alteration agreed to the negatively connotated statements compared to the no alteration group. The HNC patients in the alteration group experienced more eating difficulties such as eating in smaller portions, having food aversion, and having difficulty eating certain foods. This may consequently lead to lower food intake, as it was shown that sensory alterations were correlated with a negative impact on nutritional status [5, 10].

The present study demonstrated that HNC patients experienced several oral symptoms. The oral symptoms frequently experienced by patients were dry mouth, sticky saliva, difficulty chewing, difficulty swallowing, food stuck in the mouth, and food stuck in the throat. These symptoms seem to be mediated by the lack of salivation, as observed in our previous publication [19]. The perception of dry mouth and sticky saliva were experienced by 80% and 60% of patients, respectively. The prevalence of dry mouth and thick saliva among HNC patients who have completed radiotherapy in previous studies was approximately 90% [5, 32]. The prevalence was higher in previous studies as they were assessed at the end of their radiotherapy, whereas in this study patients were included 4 months to 5 years after the end of their radiotherapy. Xerostomia, defined as the subjective perception of dry mouth and/or sticky saliva due to reduced salivary flow, has been widely reported to be one of the most common side effects in this subpopulation of cancer [33].

Difficulty in swallowing and chewing were experienced by 67% and 57% of patients, respectively. Saliva is responsible for bolus formation during mastication, in “wetting and coating, hydration, and granulation” [34]. Lack of saliva will cause the food to be more compact and cohesive, making it more difficult to chew [35, 36]. In addition to salivation, difficulty in chewing may be influenced by age, jaw muscle activity, and use of dentures [35]. Following mastication, the bolus needs to be optimally moistened before it can be swallowed; hence, sufficient saliva is also necessary to facilitate swallowing [34, 37]. Previous studies have shown that difficulty in food processing is common among HNC patients post-radiotherapy, ranging from 88 to 90% for swallowing difficulty and 40 to 63% for chewing difficulty [5, 32, 38]. These altogether may lead to fear of eating due to the risk of choking [39].

Food sticking in the throat and mouth was experienced by 57% and 53% of patients, respectively. These, too, can be associated with salivary function. The hydrating and lubricating properties of saliva facilitate oral clearance [40]; therefore, the lack of it causes food to get stuck in the mouth and/or throat. The other oral symptoms that the HNC patients in this study frequently experienced were dental problems, sensitive teeth/gum, pain in the throat, and pain/problems with teeth. These symptoms can be related to salivary function, as saliva protects teeth and oro-oesophageal mucosa [40]. It was shown that pain surrounding the oral cavity was one of the symptoms reported by HNC patients associated with cancer treatments such as radiotherapy and chemotherapy [4, 5]. Further, it was suggested that severe oral symptoms may influence patients’ physical functioning, quality of life, and nutritional status [41].

The correlation between oral symptoms and sensory perception was observed. Oral symptoms, such as difficulty in chewing and swallowing, food getting stuck in the mouth, and pain in the oral cavity, were positively correlated with texture and temperature perception. It implies that patients affected by these oral symptoms exhibit increased awareness or caution when selecting foods, aiming to avoid food textures and temperatures that may cause pain or discomfort upon consumption.

In addition to the aforementioned oral symptoms, dry mouth, and oral inflammation were also correlated to the preference for sourness, spiciness, astringency, carbonation, and alcohol. Saliva serves multiple functions, including sensory perception, food oral processing, and digestion [39]; hence, impairment in salivary production may influence their eating experience and food intake. A previous study demonstrated that salivary quantity was related to the perception of oral comfort, depending on the food products. The food needs enough moisture, or compensated with some fat, to be easily processed and ingested [42]. The amount and composition of saliva influence the perception of food texture [43]. Further, the interaction between salivary protein and polyphenols influenced the perception of astringency [44, 45], whereas spiciness will become an irritating sensation with the presence of oral pain and inflammation.

Finally, the correlations between oral symptoms and eating behaviour also demonstrated that patients with more oral symptoms have more difficulty in eating situations. Notably, patients with more oral symptoms were correlated with having less appetite, eating smaller portions, not feeling at ease when eating out, not liking food before tasting, developing food aversion, and making certain foods unpleasant or difficult to eat. Consequently, it was reported in previous studies that patients with more serious oral symptoms had reduced intake and higher weight loss [4, 5]. Therefore, both sensory alterations and oral symptoms may affect patients’ eating experience, contributing to adverse nutritional and health outcomes.

Prior research has identified discrepancies between objective and self-reported measurements of sensory alterations. In particular, self-reported taste alterations tend to be overestimated, whereas subjective smell alterations tend to be underestimated [13, 14]. Relying solely on objective measurements may underestimate the complex and subjective nature of the eating experience. Patients reported altered somatosensory perception, consistent with altered somatosensory measures observed previously [19]. However, it is important to note that the results obtained from these two measurements cannot be directly compared. The objective measurements captured the current situation, while the self-reported sensory perception was captured in a retrospective manner (i.e. “in comparison to before the cancer treatment, my sensitivity has”).

This study has limitations, including its small sample size and cross-sectional design; therefore, it cannot infer causation. As the sensory perception was based on retrospective response, it would have higher validity if conducted in a longitudinal design comparing the perception before the cancer treatments and a few time points following the treatments. Moreover, data on oral health status (e.g. number of teeth, occlusal functional units) and use of palliative care (e.g. artificial saliva), which may influence food perception and eating behaviour, was not assessed. However, the study still indicates that patients perceived somatosensory alteration, together with adverse oral symptoms, as being related to greater eating difficulties, which can potentially lead to deteriorated nutritional outcomes.

## Conclusions

Eating is a fundamental act that not only fulfills physiological needs but also carries psychological value. The primary findings of the present study showed that more than half of the HNC patients perceived sensory alterations, including their somatosensory perception. These alterations were associated with different aspects of eating including sensory-related food preference and eating behaviour. In addition, common oral symptoms related to salivary dysfunction were reported by patients, which also influenced their eating experience. Patients with perceived sensory alterations and oral symptoms were more likely to face challenges in eating. In order to develop holistic nutritional interventions that enhance patients’ eating experience, it is necessary to consider these two aspects.

### Supplementary Information

Below is the link to the electronic supplementary material.

Supplementary file1 (DOCX 112 KB)

## Data Availability

The data that support the findings of this study are not openly available due to reasons of sensitivity and are available from the corresponding author upon reasonable request.

## References

[1] Burges Watson DL, Lewis S, Bryant V (2018). Altered eating: a definition and framework for assessment and intervention. BMC Nutr.

[2] Citak E, Tulek Z, Uzel O (2019). Nutritional status in patients with head and neck cancer undergoing radiotherapy: a longitudinal study. Support Care Cancer.

[3] Muscaritoli M, Molfino A, Scala F (2019). Nutritional and metabolic derangements in Mediterranean cancer patients and survivors: the ECPC 2016 survey. J Cachexia Sarcopenia Muscle.

[4] Farhangfar A, Makarewicz M, Ghosh S (2014). Nutrition impact symptoms in a population cohort of head and neck cancer patients: multivariate regression analysis of symptoms on oral intake, weight loss and survival. Oral Oncol.

[5] Wang Y, Lu Q, Zhang L (2021). Nutrition impact symptom clusters in patients with head and neck cancer receiving concurrent chemoradiotherapy. J Pain Symptom Manage.

[6] Liu D, Deng Y, Sha L (2017). Impact of oral processing on texture attributes and taste perception. J Food Sci Technol.

[7] Pulito C, Cristaudo A, La PC (2020). Oral mucositis: the hidden side of cancer therapy. J Exp Clin Cancer Res.

[8] Riantiningtyas RR, Carrouel F, Bruyas A (2023). Oral somatosensory alterations in head and neck cancer patients—an overview of the evidence and causes. Cancers (Basel).

[9] Dornan M, Semple C, Moorhead A (2022). Experiences and perceptions of social eating for patients living with and beyond head and neck cancer: a qualitative study. Support Care Cancer.

[10] Hutton JL, Baracos VE, Wismer WV (2007). Chemosensory dysfunction is a primary factor in the evolution of declining nutritional status and quality of life in patients with advanced cancer. J Pain Symptom Manage.

[11] Nolden AA, Hwang LD, Boltong A, Reed DR (2019) Chemosensory changes from cancer treatment and their effects on patients’ food behavior: a scoping review. Nutrients 11:. 10.3390/nu11102285 10.3390/nu11102285 PMC6836020 31554217

[12] van Elst JM, IJzerman NS, Mathijssen RHJ (2022). Taste, smell and mouthfeel disturbances in patients with gastrointestinal stromal tumors treated with tyrosine-kinase inhibitors. Support Care Cancer.

[13] Gunn L, Gilbert J, Nenclares P (2021). Taste dysfunction following radiotherapy to the head and neck: a systematic review. Radiother Oncol.

[14] Álvarez-Camacho M, Gonella S, Campbell S (2017). A systematic review of smell alterations after radiotherapy for head and neck cancer. Cancer Treat Rev.

[15] McLaughlin L, Mahon SM (2014) Taste dysfunction and eating behaviors in survivors of head and neck cancer treatment. Medsurg Nurs 23:165–184 25137792

[16] Crowder SL, Najam N, Sarma KP (2020). Head and neck cancer survivors’ experiences with chronic nutrition impact symptom burden after radiation: a qualitative study. J Acad Nutr Diet.

[17] Lundström JN, Boesveldt S, Albrecht J (2011). Central processing of the chemical senses: an overview. ACS Chem Neurosci.

[18] Engelen L, de Wijk RA (2012) Oral processing and texture perception. In: Chen J, Engelen L (eds) Food oral processing: fundamentals of eating and sensory perception. John Wiley & Sons, Incorporated. pp 159–176. 10.1002/9781444360943.ch8

[19] Riantiningtyas RR, Valenti A, Dougkas A (2023). Oral somatosensory alterations and salivary dysfunction in head and neck cancer patients. Support Care Cancer.

[20] Amézaga J, Alfaro B, Ríos Y (2018). Assessing taste and smell alterations in cancer patients undergoing chemotherapy according to treatment. Support Care Cancer.

[21] de Haan JJ, Renken RJ, Moshage Y (2021). Self-reported taste and smell alterations and the liking of oral nutritional supplements with sensory-adapted flavors in cancer patients receiving systemic antitumor treatment. Support Care Cancer.

[22] Drareni K, Bensafi M, Giboreau A, Dougkas A (2021). Chemotherapy-induced taste and smell changes influence food perception in cancer patients. Support Care Cancer.

[23] Hunot C, Fildes A, Croker H (2016). Appetitive traits and relationships with BMI in adults: development of the adult eating behaviour questionnaire. Appetite.

[24] Singer S, Amdal CD, Hammerlid E (2019). International validation of the revised European organisation for research and treatment of cancer head and neck cancer module, the EORTC QLQ-HN43: Phase IV. Head Neck.

[25] Kolde R (2015) Pheatmap: pretty heatmaps. R package version 1.0.12. 1–7. https://github.com/ReisyaRR/Sensory-perception-cancer/tree/main#create-heatnap-of-hierarchical-cluster-analysis-on-r

[26] Galaniha LT, Nolden AA (2023) Characteristics and extent of taste alterations in cancer patients during and after treatments and potential taste alteration management strategies. In: Pangborn Sensory Science Sympsosium. 20–24 August 2023. Nantes, France

[27] Drareni K (2020) Taste and cancer: satisfy the senses to maintain food enjoyment during chemotherapy. PhD Thesis. University Claude Bernard Lyon 1. Available at: https://theses.fr/2020LYSE1008

[28] IJpma I, Renken RJ, Ter Horst GJ, Reyners AKL (2016). The palatability of oral nutritional supplements: before, during, and after chemotherapy. Support Care Cancer.

[29] Boltong A, Campbell K (2013). “Taste” changes: a problem for patients and their dietitians. Nutr Diet.

[30] Dalton J, Rothpletz-Puglia P, Epstein JB (2022). Transitioning the eating experience in survivors of head and neck cancer. Support Care Cancer.

[31] Ganzer H, Touger-Decker R, Byham-Gray L (2015). The eating experience after treatment for head and neck cancer: a review of the literature. Oral Oncol.

[32] Jin S, Lu Q, Sun Y (2021). Nutrition impact symptoms and weight loss in head and neck cancer during radiotherapy: a longitudinal study. BMJ Support Palliat Care.

[33] Vistoso Monreal A, Polonsky G, Shiboski C (2022). Salivary gland dysfunction secondary to cancer treatment. Front Oral Heal.

[34] Guo Q (2021). Understanding the oral processing of solid foods: insights from food structure. Compr Rev Food Sci Food Saf.

[35] van der Bilt A, Chen J, Engelen L (2012). Oral management of food. Food oral processing: fundamentals of eating and sensory perception.

[36] Logemann JA, Smith CH, Pauloski BR (2001). Effects of xerostomia on perception and performance of swallow function. Head Neck.

[37] Liu D, Deng Y, Sha L, et al Impact of oral processing on texture attributes and taste perception. J Food Sci Technol 54:. 10.1007/s13197-017-2661-1 10.1007/s13197-017-2661-1 PMC5502015 28740316

[38] Langius JAE, Doornaert P, Spreeuwenberg MD (2010). Radiotherapy on the neck nodes predicts severe weight loss in patients with early stage laryngeal cancer. Radiother Oncol.

[39] Pedersen AML, Sørensen CE, Proctor GB, Carpenter GH (2018). Salivary functions in mastication, taste and textural perception, swallowing and initial digestion. Oral Dis.

[40] Pedersen AM, Bardow A, Jensen SB, Nauntofte B (2002). Saliva and gastrointestinal functions of taste, mastication, swallowing and digestion. Oral Dis.

[41] Crowder SL, Douglas KG, Yanina Pepino M (2018). Nutrition impact symptoms and associated outcomes in post-chemoradiotherapy head and neck cancer survivors: a systematic review. J Cancer Surviv.

[42] Assad-Bustillos M, Tournier C, Septier C (2019). Relationships of oral comfort perception and bolus properties in the elderly with salivary flow rate and oral health status for two soft cereal foods. Food Res Int.

[43] Engelen L, van den Keybus PAM, de Wijk RA (2007). The effect of saliva composition on texture perception of semi-solids. Arch Oral Biol.

[44] De Wijk RA, Prinz JF (2006). Mechanisms underlying the role of friction in oral texture. J Texture Stud.

[45] Dinnella C, Recchia A, Fia G (2009). Saliva characteristics and individual sensitivity to phenolic astringent stimuli. Chem Senses.

