# Supplementary material for: Investigating oral somatosensory perception and oral symptoms of head and neck cancer patients: insights on eating behaviour
Source: Support Care Cancer. 2024 May 1;32(5):320. doi: 10.1007/s00520-024-08512-4 (PMC11062985; doi:10.1007/s00520-024-08512-4)
Supplement: Supplementary file 1 — Supplementary file1 (DOCX 112 KB) [file 520_2024_8512_MOESM1_ESM.docx]

**Supplementary material S1 – Questionnaire**

Q1.1
**Sensory** **sensitivity**
The following questions are similar for each sense studied. We ask you to answer according to your **SENSITIVITY** and not your appetite/preference.

Q1.2.1

**TASTE**
Compared to the situation before cancer treatment:

|  | Strongly disagree | Disagree | Somewhat disagree | Somewhat agree | Agree | Strongly agree |
| --- | --- | --- | --- | --- | --- | --- |
| I notice changes in the taste of food/drinks |  |  |  |  |  |  |

Q1.2.2

**Compared to the situation before the cancer treatment, I perceive that my sensitivity to:**

|  | Decreased | Unchanged | Increased |
| --- | --- | --- | --- |
| ...salty products |  |  |  |
| ...sweet products |  |  |  |
| ...sour products |  |  |  |
| ...bitter products |  |  |  |
| ...umami* products |  |  |  |

* Umami: is the fifth taste detected by the tongue, after salty, sweet, sour and bitter. Generally translated as "savoury taste", umami corresponds to the taste of sodium mono-glutamate.

Here is a non-exhaustive list of ingredients very rich in umami: ripe tomatoes, mature cheeses (Roquefort, Camembert, Cheddar, etc.), dried mushrooms (shiitake, morels, etc.), soy sauce, anchovies, asparagus, dried bonito, cold meats, onions, truffles, wakame seaweed, kombu.

Q1.3

**SMELL**

**Compared to the situation before the cancer treatment, I perceive that my sensitivity to:**

|  | Decreased | Unchanged | Increased | Different |
| --- | --- | --- | --- | --- |
| ...smell of food/drinks |  |  |  |  |

Q1.4
**TEXTURE**

 Compared to the situation before cancer treatment:

|  | Strongly disagree | Disagree | Somewhat disagree | Somewhat agree | Agree | Strongly agree |
| --- | --- | --- | --- | --- | --- | --- |
| I have noticed changes in my perception of textures |  |  |  |  |  |  |

Q1.5

TEMPERATURE
Compared to the situation before cancer treatment:

|  | Decreased | Unchanged | Increased |
| --- | --- | --- | --- |
| My sensitivity to hot food/drinks has: |  |  |  |
| My sensitivity to cold foods/drinks has: |  |  |  |

**You are now going to answer questions about other types of oral sensations.**

 Q1.6
- ***Astringency*** refers to a sensation in the mouth most often described as "drying or rough". It is caused by a wide variety of foods and beverages, including strong teas, red wines, nuts, and various (usually unripe) fruits.
 -   ***Pungency*** is the condition of having a strong, irritating/pungent odor or flavor. This is the characteristic of foods commonly referred to as hot or pungent. It is found in foods such as chilli, peppers, mustard, garlic, arugula, wasabi.
 - ***Carbonated drinks*** are drinks containing dissolved carbon dioxide. They include sparkling water and soft drinks (e.g. lemonade, soda, coca cola, orangina, champomy, Schweppes). They do not include alcoholic beverages (e.g. champagne, beer).
 -**Alcoholic** **drinks**are beverages containing 3 to 50% alcohol (e.g. beers, wines, spirits). They do not include fermented drinks with an alcohol content < 0.5% or non-alcoholic alternatives (e.g. kombucha, low alcohol beer 0.5%).


 **Compared to the situation before the cancer treatment, I perceive that my sensitivity to:**

|  | Decreased | Unchanged | Increased |
| --- | --- | --- | --- |
| ...spicy/pungent products (e.g. chili, curry)... |  |  |  |
| ...cooling products (e.g. mint)... |  |  |  |
| ...astringent products (e.g. wine, green tea)... |  |  |  |
| ...carbonated drinks... |  |  |  |
| ...alcoholic drinks... |  |  |  |

**Now you will answer questions about your food preferences**

  Q2.1 In comparison with the situation before cancer treatment, my preference towards

|  | has decreased | no change | has increased |
| --- | --- | --- | --- |
| ... salty products |  |  |  |
| ... sweet products |  |  |  |
| ... sour products |  |  |  |
| ...bitter products |  |  |  |

Q2.2 In comparison with the situation before cancer treatment, my preference towards

|  | has decreased | no change | has increased |
| --- | --- | --- | --- |
| spicy/pungent products (e.g. chili, curry) |  |  |  |
| refreshing products (e.g. mint) |  |  |  |
| astringent products (e.g. wine) |  |  |  |
| carbonated drinks (e.g. soda, sparkling water) |  |  |  |
| Alcoholic drinks |  |  |  |

Q3.1
**You will answer questions on your eating habits**

Q3.2
**How have your eating habits changed from before treatment?**

|  | Strongly disagree | Disagree | Somewhat disagree | Somewhat agree | Agree | Strongly agree |
| --- | --- | --- | --- | --- | --- | --- |
| When I see or smell food I like, it makes me want to eat |  |  |  |  |  |  |
| I like a wide variety of foods |  |  |  |  |  |  |
| I am interested in tasting new food I haven't tasted before |  |  |  |  |  |  |
| I have less appetite |  |  |  |  |  |  |
| I get full more quickly |  |  |  |  |  |  |
| I eat in smaller portions |  |  |  |  |  |  |
| I eat more frequently |  |  |  |  |  |  |

Q3.3 **How have your eating habits changed compared to before treatment?**

|  | Strongly disagree | Disagree | Somewhat disagree | Somewhat agree | Agree | Strongly disagree |
| --- | --- | --- | --- | --- | --- | --- |
| Eating takes more effort |  |  |  |  |  |  |
| I lost the pleasure of eating |  |  |  |  |  |  |
| I feel uncomfortable eating outside my home |  |  |  |  |  |  |
| I am often the last to finish the meal |  |  |  |  |  |  |
| I often decide that I don't like a food before tasting it. |  |  |  |  |  |  |
| I have started to strongly dislike or avoid certain foods |  |  |  |  |  |  |
| I have a strong desire or craving to eat certain foods |  |  |  |  |  |  |

Q4.1 **You will answer questions on your oral symptoms.**

Q4.2 How frequently do you experience these symptoms?

|  | Never | Rarely | Sometimes | Often | Always |
| --- | --- | --- | --- | --- | --- |
| I have mouth sores |  |  |  |  |  |
| I limit the amount or kind of food I eat because of dental problems |  |  |  |  |  |
| I have difficulty biting or chewing certain hard foods such as meat or an apple |  |  |  |  |  |
| I have difficulty swallowing |  |  |  |  |  |
| My teeth or gums are sensitive to cold, hot or sugary foods |  |  |  |  |  |
| Food gets stuck in my mouth |  |  |  |  |  |
| Food gets stuck in my throat |  |  |  |  |  |
| I dread the moment of eating because I have pain |  |  |  |  |  |
| I am limited in my ability to open or move my jaw |  |  |  |  |  |

Q4.3

|  | Never | Rarely | Sometimes | Often | Always |
| --- | --- | --- | --- | --- | --- |
| I have a dry mouth |  |  |  |  |  |
| I have sticky saliva |  |  |  |  |  |
| I feel nauseous |  |  |  |  |  |
| I have pain on my gums |  |  |  |  |  |
| I have bleeding gums |  |  |  |  |  |
| I have sore lips |  |  |  |  |  |
| I have a sore mouth |  |  |  |  |  |
| I have a burning sensation in my mouth |  |  |  |  |  |
| I have a pain in my throat |  |  |  |  |  |
| I have dental pain/ problems |  |  |  |  |  |

# **Supplementary material S2 – Correlation tables**


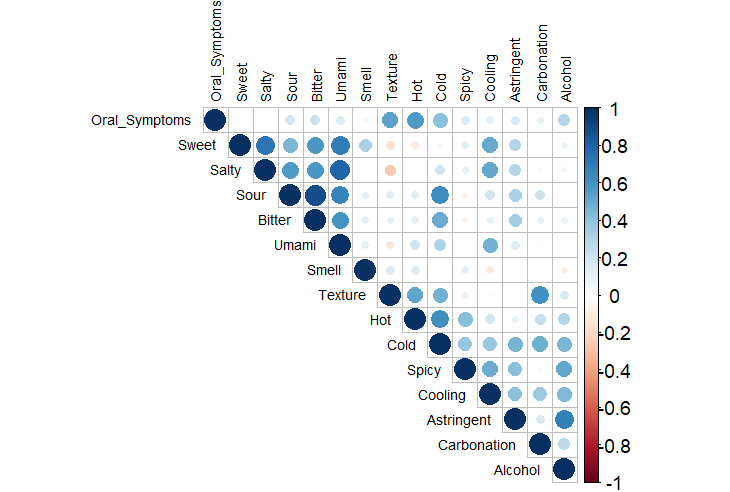


**Supplementary Figure 1.** Correlations between oral symptoms and sensory perception


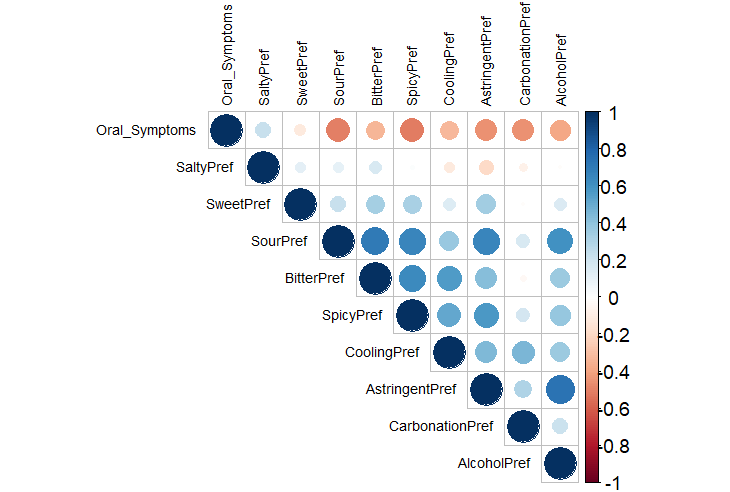


**Supplementary Figure 2.** Correlations between oral symptoms and food-related sensory preferences


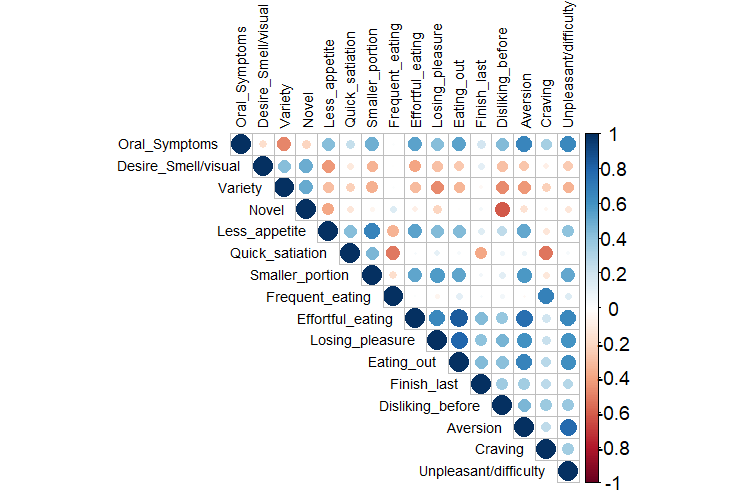


**Supplementary Figure 3.** Correlations between oral symptoms and eating behaviour
